# Bovine innate immune phenotyping via a standardized whole blood stimulation assay

**DOI:** 10.1038/s41598-021-96493-3

**Published:** 2021-08-26

**Authors:** Cian Reid, Charlotte Beynon, Emer Kennedy, Cliona O’Farrelly, Kieran G. Meade

**Affiliations:** 1 Animal and Bioscience Research Department, Animal & Grassland Research and Innovation Centre, Teagasc, Grange, Co Meath Ireland; 2 School of Biochemistry and Immunology, Trinity College Dublin, Dublin 2, Ireland; 3 Teagasc, Animal & Grassland Research and Innovation Centre, Moorepark, Fermoy Co. Cork, Ireland; 4 School of Medicine, Trinity College Dublin, Dublin 2, Ireland; 5 School of Agriculture and Food Science, University College Dublin, Belfield, Dublin 4, Ireland; 6 Conway Institute of Biomolecular and Biomedical Research, University College Dublin, Belfield, Dublin 4, Ireland; 7 Institute of Food and Health, University College Dublin, Belfield, Dublin 4, Ireland

**Keywords:** Biological techniques, Immunology, Applied immunology, Innate immunity

## Abstract

Cattle vary in their susceptibility to infection and immunopathology, but our ability to measure and longitudinally profile immune response variation is limited by the lack of standardized immune phenotyping assays for high-throughput analysis. Here we report longitudinal innate immune response profiles in cattle using a low-blood volume, whole blood stimulation system—the ImmunoChek (IChek) assay. By minimizing cell manipulation, our standardized system minimizes the potential for artefactual results and enables repeatable temporal comparative analysis in cattle. IChek successfully captured biological variation in innate cytokine (IL-1β and IL-6) and chemokine (IL-8) responses to 24-hr stimulation with either Gram-negative (LPS), Gram-positive (PamCSK4) bacterial or viral (R848) pathogen-associated molecular patterns (PAMPs) across a 4-month time window. Significant and repeatable patterns of inter-individual variation in cytokine and chemokine responses, as well as consistent high innate immune responder individuals were identified at both baseline and induced levels. Correlation coefficients between immune response read-outs (IL-1β, IL-6 and IL-8) varied according to PAMP. Strong significant positive correlations were observed between circulating monocytes and IL-6 levels for null and induced responses (0.49–0.61) and between neutrophils and cytokine responses to R848 (0.38–0.47). The standardized assay facilitates high-throughput bovine innate immune response profiling to identify phenotypes associated with disease susceptibility and responses to vaccination.

## Introduction

The innate immune system plays a critical role in the early immune response and determines outcome to bacterial and viral infections^1^, making it an obvious starting point for the identification of immune phenotypes associated with disease susceptibility. A rapid, efficient innate response can potentially clear infection without activation of adaptive immune mechanisms and results in minimal pathology. Due to its non-specific nature, an efficient innate immune response could also offer cross protection against diverse infections^2^. Innate immune cells have multiple anti-pathogen effector mechanisms, including phagocytosis, cytokine secretion, the production of reactive oxygen species (ROS) and antimicrobial peptides^3^. Cytokines and chemokines, including Interleukin 1 (IL-1), Interleukin 6 (IL-6) and Interleukin 8 (IL-8) are key modulators of the innate response—involved in recruiting immune cells to the site of infection and activating pathogen-killing mechanisms, often leading to inflammation^4,5^. Certain other cytokines (e.g. IL-10) can also modulate resolution of the inflammation, to prevent excessive or persistent inflammation that contributes to pathology^6^. Therefore, cytokines and chemokines are immune response sentinels, and the ratio between these molecules has important relevance for the course of the immune response and ultimately the ability of the host to counter infection. Additionally, many cytokines also modulate adaptive immune responses—both directly and indirectly^7^. Therefore, innate immune response profiling can also identify successful cross talk between innate and adaptive immune responses, which will ultimately influence the development of protective immunity and thereby the success of vaccination.

Cytokine profiling studies in humans has revealed significant inter-individual variation in both ‘basal’ and induced cytokine and chemokine responses as well as in the dynamics of the immune response over time. Standardized whole blood stimulation assays (*TruCulture*) were used to define the variability of immune activity in humans, via measurement of immune responses in 25 healthy individuals to 27 different immune stimuli including cytokines and chemokines^8^. Significant inter-individual variation was apparent in both basal and induced responses which is likely to have important relevance for disease susceptibility. Two individuals lacked a detectable IL-1α response to lipopolysaccharide (LPS), which was postulated to have potential disease-relevance and such information in cattle would also have broader relevance in terms of improving vaccination strategies^9^. The individuals used in this study are also part of a larger cohort in which detailed health traits have been recorded for each individual in order to assess the risk factors involved in disease susceptibility and to identify the key contributors to human immunological variance^10^. Already, cytokine signatures found using these assays have been linked with disease—including diabetes^11^, and enabled the discrimination between clinical and latent TB^12^. Impaired cytokine responses to infection also leads to increased disease severity, most recently shown in COVID-19 patients^13^, showing additional disease relevance.

In livestock, disease-associated changes in cytokine responses have not been as extensively explored. However, like humans, cattle are outbred with significant inter-individual variation in susceptibility to infection and immune pathology. A large degree of inter-individual variation has been shown in clinical, biochemical, hematological and immune parameters^14,15^, which have important relevance for disease susceptibility and responses to vaccination. Many studies have also now shown cytokine profiles to have disease relevance. Cattle infected with bovine viral diarrhoea virus (BVDV) showed reduced levels of IL-1β and IL-10, correlating with a more severe secondary infection of bovine herpes virus 1 (BHV1)^16^. Elevated baseline levels of the chemokine IL-8 was associated with resistance to reproductive and respiratory syndromes in pigs^17^, showing basal levels of immune proteins can have an important role in protection against disease. However, the majority of these studies were performed in small numbers of animals where inter-individual variation cannot be comprehensively studied.

Current approaches to assessing immune responses in livestock require detailed laboratory protocols for cell separation and stimulation experiments, which can hamper comparisons between studies or across time. Additionally, these methods can also invariably normalize the induced immune response to a control or null response, precluding the analysis of unstimulated or ‘basal’ expression. Significant differences in methodology between laboratories have the potential to introduce artefactual errors into results, as shown in a study in humans examining cytokine responses after PBMC stimulation^18^. It found that PBMC stimulations had higher technical and biological variation compared to the standardized whole blood stimulation assay *TruCulture*. It was concluded that *TruCulture*’s use of whole blood produced a more accurate representation of individual immune response’s due to the inclusion of multiple cell type interactions, and it being a standardized method which reduced technical error and variation between operators. Our ability to longitudinally profile immune responses in cattle, particularly in any high-throughput manner is limited by the lack of standardised assays similar to that used in human studies. Our previous study applied the *TruCulture* system to immune-profiling in calves^19^, but cost and proprietary aspects of these commercial assays preclude widespread use in agriculture. Additionally, species-specific differences in immune responses was apparent, particularly in response to the viral PAMP Poly:IC which did not induce an immune response in cattle.

In this study, we longitudinally profile the innate immune responses of adult cattle to bacterial and viral PAMPs using a novel, low cost, and low volume whole blood stimulation assay called Immuno-Chek (IChek) that can measure informative immune phenotypes in cattle. As experimental infection studies are expensive and have obvious animal welfare drawbacks, high throughput immune profiling that can identify natural immune variation could facilitate targeted management, earlier interventions, reduction in antimicrobial usage contributing to antimicrobial resistance and potentially enhance animal selection practices.

## Results

### Optimization of PAMP concentrations

Initially, different concentrations of PAMPs were tested to determine the optimal concentration to induce a robust response in bovine whole blood. The immune response readouts used were protein levels of the cytokines IL-1β, IL-6 and the chemokine IL-8.

**Figure 1:**
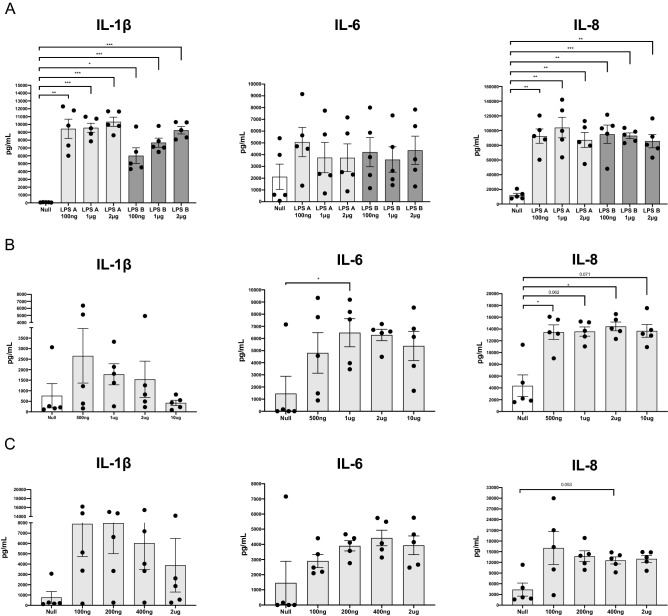
Dose responses of bacterial and viral PAMP using IChek. Dose responses of five animals stimulated for 24 hr with (**A**) 0.1–2 µg/mL of LPS serotype A and B; (**B**) 0.5-10 µg/mL of Pam3CSK4 and (**C**) 0.1–2 µg/mL of R848 or null, using 1 mL of whole blood collected for each stimulation. Protein expression of IL-1β, IL-8 and IL-6 was measured in the supernatant of each stimulation using ELISA, reported as pg per 1 mL of blood, and were used as immune response readouts. Statistical significance was calculated using repeated measures one-way ANOVA with Dunnett’s multiple comparisons between null and induced responses.

Responses to two different LPS serotypes were examined, from *E. coli* strains O111:B4 (LPS A) and O55:B5 (LPS B). Clear significant induction of both IL-1β and IL-8 protein were detected after 24 hr in response to all LPS concentrations used (Fig. 1A), relative to null responses. No significant difference in the immune response was detected between the two LPS serotypes and IL-6 did not show any consistency in responses to either LPS serotype. 2 µg/mL of LPS serotype O111:B4 was chosen for subsequent stimulations.

Significant induction of IL-8 was observed after 24 hr in response to 0.5 µg and 2 µg/mL of Pam3CSK4 and of IL-6 in response to 1ug of Pam3CSK4 (Fig. 1B), relative to null responses. However, elevated levels IL-1β and IL-8 in response to 1 μg/mL of Pam3CSK4 were observed in three animals and four animals, respectively. On the basis of these results, 1 µg/mL of Pam3CSK4 was chosen for subsequent stimulations.

No significant difference was observed after 24 hr in response R848 for any concentrations (Fig. 1C), relative to null responses. However, clear induction was observed for IL-1β, IL-6 and IL-8 for 3, 4 and 4 animals respectively, in response to 0.2 µg/mL of R848. On the basis if these results, 0.2 µg/mL of R848 was chosen for subsequent stimulations.

### Longitudinal and inter-individual variation in immune cell populations

**Figure 2:**
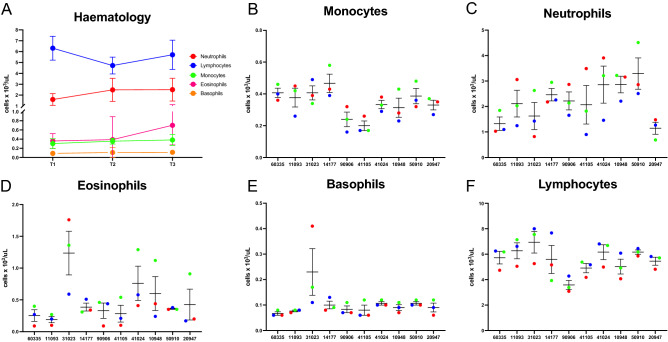
Absolute cell counts for monocytes, neutrophils, eosinophils, basophils and lymphocytes, reported as cells × 10^3^/µL, using blood collected in EDTA tubes from ten cattle at three timepoints across 4 months. (**A**) Haematology data shown for the whole group of cattle (n = 10) across three time points. (**B**–**F**) Individual haematology data for each animal at three timepoints (blue = timepoint 1, red = timepoint 2, green = timepoint 3). Results were graphed using Prism software (v9.1) (www.graphpad.com).

To control for differences in immune protein expression that could result from sub-clinical disease, hematology data was collected for all animals at each time point. Monocyte, neutrophil, eosinophil, basophil and lymphocyte populations were assessed with average absolute circulating numbers of cells reported for the group across the three time points (Fig. 2A). Lymphocytes were the most abundant cell population in whole blood, followed by neutrophils.

At an individual animal level, large inter-individual and temporal changes in cell populations were evident across time for all cell populations (Fig. 2B–F). Most notably, time point 2 displays the largest magnitude of differences between individuals for absolute counts. Additionally, neutrophil absolute cell counts display the largest temporal changes within individuals. Furthermore, based on published reference values all animals were within what are considered healthy ranges^20^.

### Qualitative and quantitative analysis of the natural inter-individual variation in the bovine innate immune response

**Figure 3:**
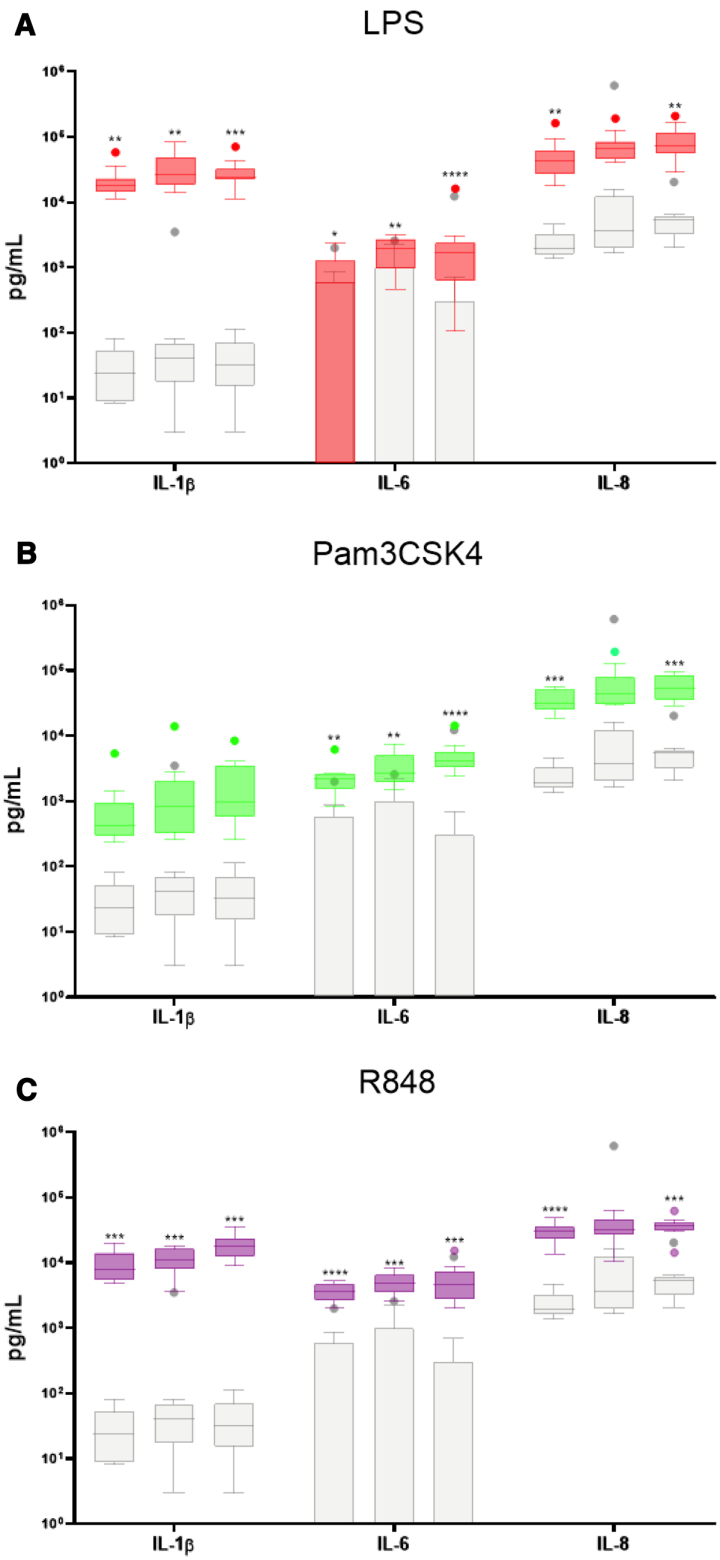
Inter-individual variation of cytokine responses to bacterial and viral PAMPs using IChek. Box and whisker plots of induced responses for ten animals at three timepoints to (**A**) 2 µg/mL of LPS (red), (**B**) 1 µg/mL of Pam3CSK4 (green) and (**C**) 0.2 µg/mL of R848 (purple), with each graph overlaid with the null levels in grey, using 1 mL blood for each 24 hr stimulation. Protein expression of IL-1β, IL-8 and IL-6 was measured in the supernatant of each stimulation using ELISA, reported as pg per 1 mL of blood, and were used as immune response readouts. The boxes represent the interquartile range with the line representing the median. The whiskers represent the interquartile range multiplied by 1.5, and dots represent the outliers. Each immune readout in graphs A-C labels three boxes, which sequentially represent timepoints 1, 2 and 3. Statistical significance was calculated using two-way ANOVA with Dunnett multiple comparisons between null and induced responses. Results were graphed using Prism software (v9.1) (www.graphpad.com).

IChek assay was performed on ten animals across three time points for all four conditions (Null stimulation, 2 µg/mL of LPS, 1 µg/mL Pam3CSK4 and 1 µg/mL R848). Figure 3 shows the overall variation in immune cytokine responses found between the ten animals across three timepoints. Significantly elevated responses were evident for IL-1β, IL-6 and IL-8 responses to bacterial LPS (Fig. 3A), Pam3CSK4 (Fig. 3B) and viral PAMP R848 (Fig. 3C). Induced responses were up to 8000-fold increased compared to the unstimulated levels. Furthermore, inter-individual variation was observed in the null response for all three immune readouts.

The inter-individual variation in immune response readouts was calculated for each PAMP, represented by a coefficient of variation (CV) between all individuals at each time point, shown in Table 1. For the null responses, IL-6 showed the highest variation and time point 2 showed the highest variation for IL-1β and IL-8. For the LPS responses, IL-6 again showed the highest variation and IL-1β showed the lowest average CV for the three timepoints. For Pam3CSK4 responses, IL-1β showed the highest variation across the three timepoints and IL-8 showed the lowest average CV. For the R848 responses, IL-1β and IL-6 had equal average CV and IL-8 had the lowest average CV. Overall, it was found the null response had higher average CVs than the induced responses, with the exception of IL-1β in response to Pam3CSK4 having the highest average CV for IL-1β. Additionally, in response the viral ligand R848, the average CVs were the lowest for all three immune response readouts.

**Table 1:** The inter-individual variation in null and induced responses represented by CVs calculated between individuals.

Timepoint	Null CVs (%)			LPS CVs (%)			Pam3CSK4 CVs (%)			R848 CVs (%)		
	IL-1ß	IL-6	IL-8	IL-1ß	IL-6	IL-8	IL-1ß	IL-6	IL-8	IL-1ß	IL-6	IL-8
1	81	174	45	64	104	79	156	60	39	54	30	33
2	284	186	291	64	54	62	182	59	82	43	37	42
3	81	293	85	56	162	62	123	67	43	42	72	33
Average	148	218	140	61	107	68	153	62	57	46	46	36

### Defining the healthy ranges in immune responses and the identification of outliers

**Figure 4:**
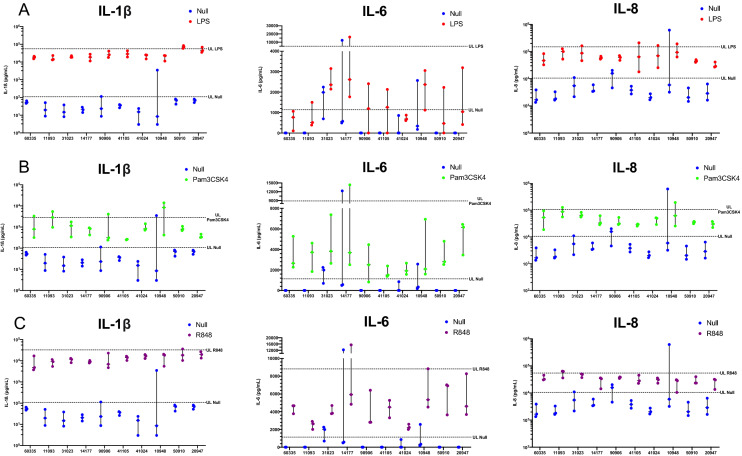
Individual responses of ten animals to PAMP or null stimulation using IChek across three timepoints to (**A**) 2 µg/mL of LPS (red), (**B**) 1 µg/mL of Pam3CSK4 (green) and (**C**) 0.2 µg/mL of R848 (purple), or null (blue), using 1 mL of blood for each 24 hr stimulation. Protein expression of IL-1β, IL-8 and IL-6 was measured in the supernatant of each stimulation using ELISA, reported as pg per 1 mL of blood, and were used as immune response readouts. Each animals individual null response is plotted alongside the induced responses, with three dots representing the response across three timepoints. The upper limits (UL) (3rd quartile + (1.5 × IQR)) for the null response and induced responses was calculated for all the data generated across the three timepoints for each immune readout and is represented by a line on each graph. Results were graphed using Prism software (v9.1) (www.graphpad.com).

The variation in null and induced responses for individual cattle is shown for all three time points in Fig. 4. Both IL-1β and IL-8 are expressed in the absence of specific stimulation, and a clear induction is evident across all animals for all three PAMPs (Fig. 4A–C). Null response levels of IL-6 are either low or not detectable, and a clear induction is also evident, although inter-individual variation in responses is most apparent with this cytokine.

**Figure 5:**
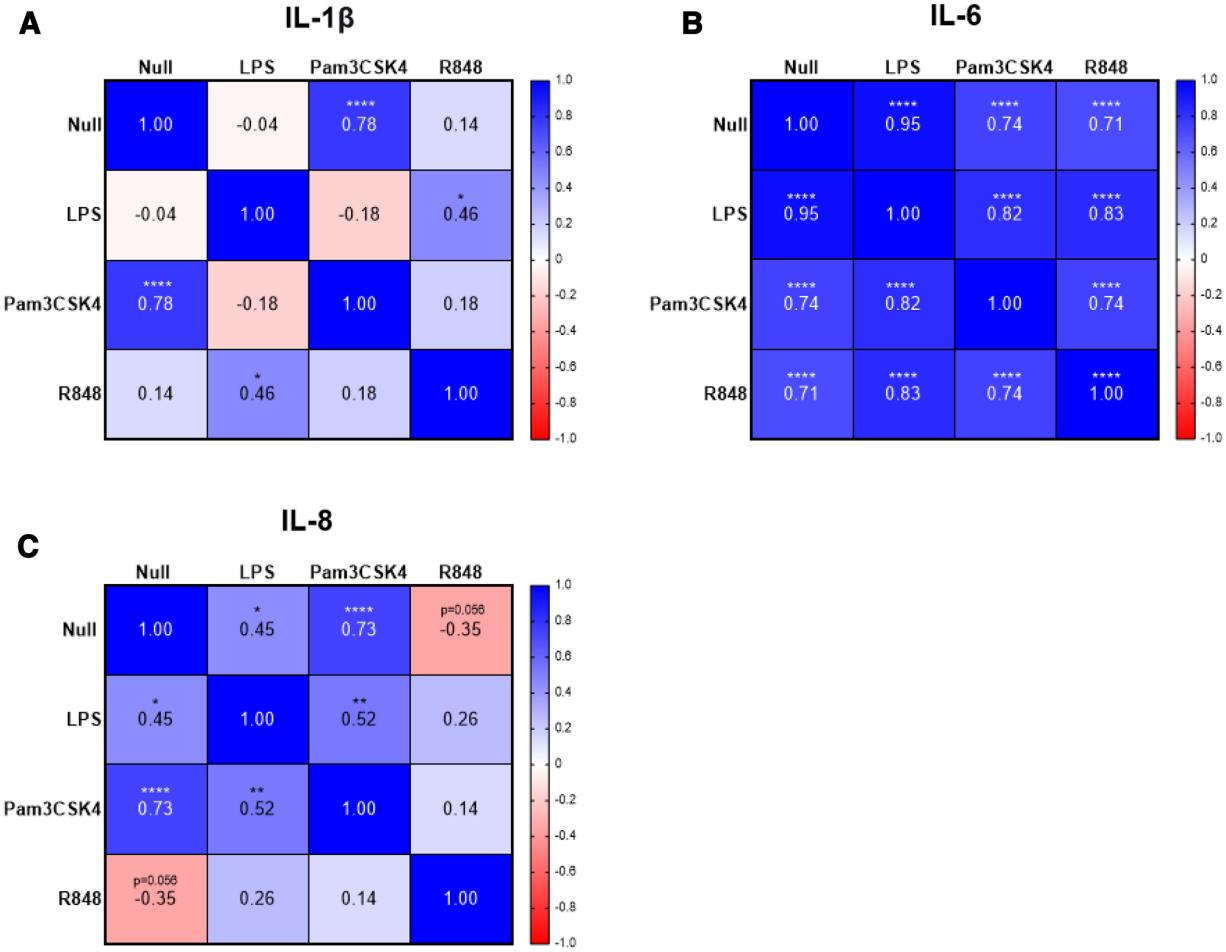
Correlation analysis of cytokine and chemokine responses to multiple PAMPs. Pearson’s correlation coefficient matrices, represented as a heatmap, calculated for (**A**) IL-1β, (**B**) IL-6 and (**C**) IL-8 between responses to null, 2 µg/mL of LPS, 1 µg/mL of Pam3CSK4, 0.2 µg/mL of R848 responses for ten animals across three timepoints. Results were graphed using Prism software (v9.1) (www.graphpad.com).

Upper and lower limits were calculated for null and induced responses for all three immune readouts to identify potential outliers with distinct immune response profiles. Animal 50910 was found consistently across the three timepoints to be above the upper limit for IL-1β in response to LPS (Fig. 4A). Additionally, animal 14177 was above the upper limit at one timepoint for IL-6 in response to LPS, whereas four animals (31023, 41105, 41024 and 10948) were identified as outliers at one timepoint each in IL-6 responses. Four animals (60335, 11093, 90906 and 10948) were identified as outliers for IL-1β in response to Pam3CSK4, with most notably animal 10948 being an outlier at two timepoints (Fig. 4B). Animal 14177 was an outlier at one timepoint for IL-6 in response to Pam3CSK4, and two animals (11093 and 10948) were identified as outliers for IL-8. Animal 50910 and 14177 were found above the upper limit at one timepoint each in response to R848 for IL-1β and IL-6 respectively, whereas animal 11093 was found above the upper limit for two timepoints for the IL-8 response (Fig. 4C). Furthermore, at one time point one animal (10948) shows above the upper limit for null responses for all three immune readouts (Fig. 4A–C). Additionally, animal 31023 and 14177 were above the upper limits for one and two timepoints for IL-6, respectively for null responses, along with animal 31023 and 11093 above the upper limits for one and two timepoints respectively for IL-8 responses. No animals were found below the lower limit for induced or null responses in this study and are not indicated in Fig. 5.

### Cytokine and chemokine responses are correlated between PAMPs

To determine whether individual cytokine and chemokine responses shown in Fig. 4, were consistent across different PAMPs, a correlation matrix was performed for each immune readout (Fig. 5A–C). For IL-1β, significant moderate to strong positive correlations were found between null and Pam3CSK4 responses (0.78), and between LPS and R848 responses (0.46) (Fig. 5A). For IL-6, significant strong correlations were identified between all responses (all > 0.7) (Fig. 5B). For IL-8, significant moderate to strong positive correlations were identified between null and LPS responses (0.45), between null and Pam3CSK4 responses (0.73), and also between Pam3CSK4 and LPS responses (0.52). In contrast, a moderate negative correlation was found between null and R848 responses (− 0.35) (Fig. 5C).

Furthermore, correlation analysis was also performed between immune readouts for each PAMPs. We detected strong correlations between IL-1β and IL-8 responses to Pam3CSK4 (0.76), and between IL-1β and IL-8 for null responses (0.98) (data not shown).

### The number of circulating immune cells in the blood prior stimulation are correlated to cytokine and chemokine responses

**Figure 6:**
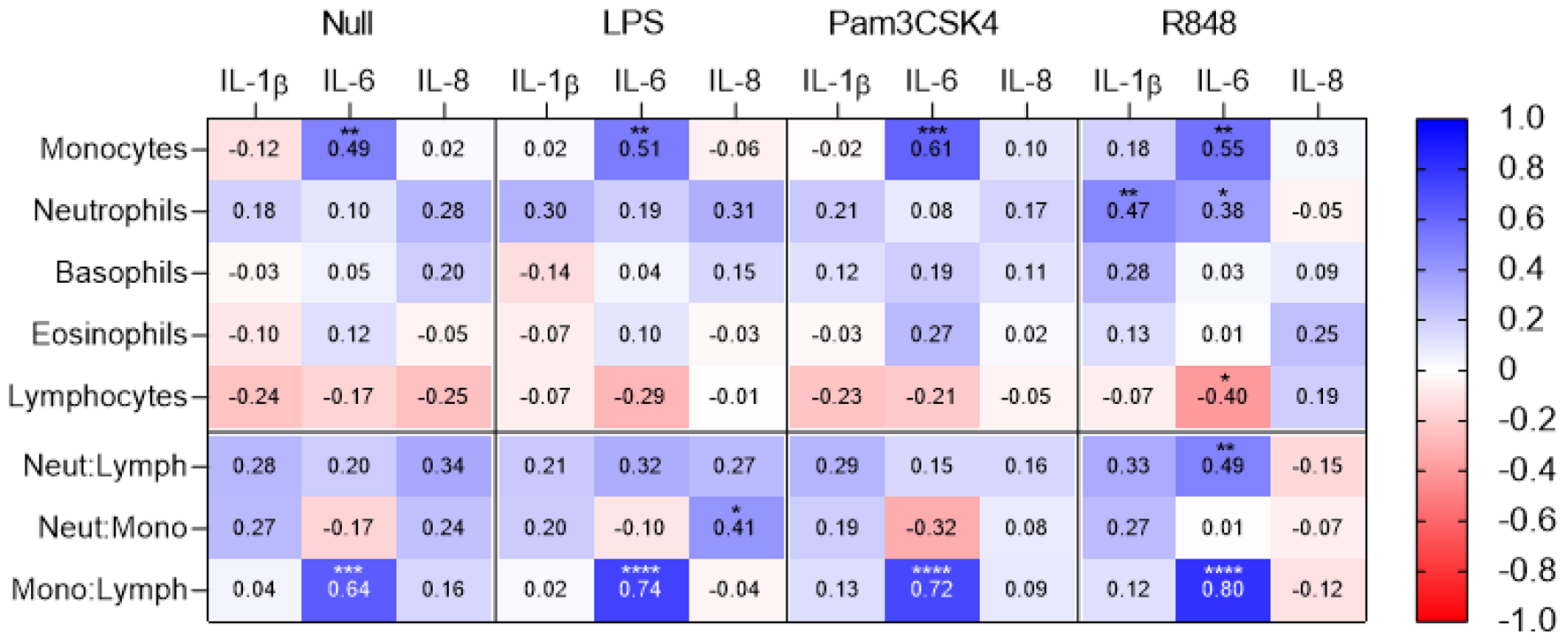
Correlations between cytokine and chemokine responses and absolute cell counts*.* Pearson’s correlation coefficients, represented as a heatmap, calculated between IL-1β, IL-6 and IL-8 levels in response to null, 2 µg/mL of LPS, 1 µg/mL of Pam3CSK4 and 0.2 µg/mL of R848, and absolute counts data for monocytes, neutrophils, eosinophils, basophils, lymphocytes and the cell ratios of neutrophils to lymphocytes, neutrophils to monocytes and monocytes to lymphocytes, gathered from the same ten animals at the same timepoints. All protein response and absolute cell counts data was generated from the same ten animals across the same three timepoints. Results were graphed using Prism software (v9.1) (www.graphpad.com).

As changes in haematology at each sampling time point could influence cytokine responses, a correlation analysis was performed between absolute cell count data and cytokine and chemokine responses, as shown in Fig. 6. Monocyte concentrations were found consistently to have significant moderate to strong positive correlations values with the IL-6 response to the null and induced responses (0.49–0.61). Neutrophils were consistently identified to have weak positive correlations for most immune readouts across all PAMPs for IL-1β and IL-8, with one significant moderate correlation detected for IL-1β in response to R848 (0.47). Basophils were found to have weak positive correlations with IL-8 for the null response (0.20) and IL-1β for the R848 response (0.28). Eosinophils were found to have weak positive correlations with IL-6 in response to Pam3CSK4 (0.27) and IL-8 in response to R848 (0.27). Additionally, lymphocytes were found to have weak negative correlations with most immune readouts for each PAMP, with one significant moderate negative correlation identified for IL-6 in response to R848 (− 0.4).

In addition, neutrophil and lymphocyte absolute counts displayed an inverse relationship with a correlation coefficient of − 0.89, and it was found that a higher neutrophil:lymphoctye ratio is correlated with elevated cytokines and chemokine responses to these PAMPs, with one significant moderate correlation identified for IL-6 in response to R848 (0.49). Other cell ratios were also included in the analysis, identifying a higher neutrophil:monocyte in most instances correlated with elevated cytokine expression, with a significant moderate correlation identified for IL-8 in response to LPS (0.41). Additionally, monocyte:lymphocyte ratios were significantly strongly correlated with IL-6 responses for all PAMPs (0.64–0.8) (Fig. 6).

### Assessment of the technical variation of IChek

**Figure 7:**
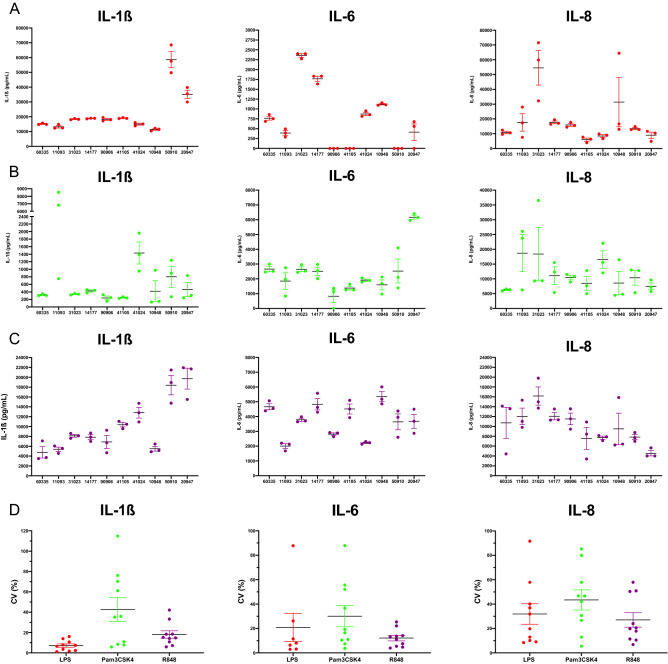
Technical variation of IChek stimulations. Individual induced responses of ten animals to (**A**) 2 µg/mL of LPS (red), (**B**) 1 µg/mL of Pam3CSK4 (green) (**C**) or 0.2 µg/mL of R848 (purple), with each stimulation carried out three times for each animal at the same time (technical replicates). Protein expression IL-1β, IL-6 and IL-8 in the supernatant of the IChek stimulations after 24 hr incubation, measured using ELISA and reported as in pg in 1 mL of blood, were used as immune readouts. (**D**) Coefficients of variation (Standard deviation/mean*100) were calculated for each immune readout between each animals technical replicates to assess the technical variation. Results were graphed using Prism software (v9.1) (www.graphpad.com).

Technical variation was assessed to evaluate the repeatability of the IChek assay, i.e. how similar the cytokine and chemokine expression levels are between three replicate stimulations for each PAMP performed at the same time point. A high degree of consistency was apparent between replicate stimulations and furthermore clearly repeatable high- and low responder cattle are evident with each PAMP, as shown in Fig. 7. The IL-1β, IL-6 and IL-8 responses for the three technical replicates for each animal in response to LPS, Pam3CSK4 and R848 are shown in Fig. 7A–C.

The coefficients of variation between the three replicates for each individual animal for these immune readouts were calculated as a representative value of the technical variation (Fig. 7D). The lowest CVs found was the IL-1β in response to LPS and remained below 20% for all ten animals. R848 also showed low CV values, with eight animals below 20% for IL-1β and all animals below 30% for IL-6. The least repeatable PAMP response was from Pam3CSK4 for all three immune readouts. The least repeatable immune readout overall was IL-8. Technical variation for null responses were not included due to low limits of detection for IL-1β and IL-6 for most animals.

## Discussion

Immunological competence is recognized as a critical determinant of life-time reproductive success and fitness in livestock species. Yet our knowledge of the immune response in livestock species lags significantly behind what we know about other traits of agricultural importance including fertility, milk yield and growth traits. The advent of new technologies has enabled a more detailed characterization of the architecture of the immune response in humans enabling identification of susceptibility and disease-associated immune response profiles^12^. Specifically, in terms of profiling cytokine responses, commercial whole blood stimulation systems facilitate longitudinal profiling of responses in the absence of stimulation as well as in response to disease-relevant PAMPs^8^. Identification of parameters which define a ‘normal’ immune response can be defined in healthy individuals which can then be used as a baseline to identify outliers which may be at risk of developing disease.

Similar approaches in cattle would offer the potential to capture phenotypic variation in immune response profiles and identify maladaptive immune responses. In cattle, whole blood culture (Bovigam) is used commonly for the measurement IFN-γ in response to mycobacterial antigens and is specifically used for TB diagnosis^21^. However, the boundaries and temporal transitions of the immune response in healthy cattle have not been comprehensively characterized across multiple disease relevant PAMPs. Furthermore, identification of immune phenotypes associated with disease susceptibility would facilitate targeted therapeutic interventions or improved management strategies. The IChek therefore represents a novel standardized method to facilitate high-throughput innate immune response profiling in cattle.

Three disease-relevant PAMPs were selected for initial optimization in this study—chosen to represent infectious agents of relevance to cattle health. Two serotypes of bacterial LPS were analyzed, both TLR4 activators, and mimic responses to Gram negative bacterial infection, such as *E. coli,* which can cause gastro-intestinal diseases. Pam3CSK4 is a TLR1/2 activator and mimics responses to Gram positive infections, such as the *Clostridium species,* that can cause severe diarrhea or other diseases such as blackleg*.* Furthermore, R848 is a TLR7/8 activator, and mimics responses of single-stranded RNA viruses, such as bovine respiratory syncytial virus, bovine parainfluenza virus 1, bovine coronavirus and bovine viral diarrhea virus. Bovine respiratory disease is of particular consequence in young calves and is the highest cause of morbidity in Europe and the USA^22^.

Cytokines are master regulators of the immune response^23^. IL-1β, IL-6 and the chemokine IL-8 are critical for immune cell recruitment and the activation of inflammation. IL-1β is controlled by inflammasome activation and excessive or prolonged expression has been shown to precede the development of disease in cattle^24^. IL-6 is potent activator of the acute phase protein response, with exacerbated responses also being identified in many inflammatory diseases^25,26^. IL-8 is a potent neutrophil chemoattractant and has important disease relevance for multiple infections in cattle, including respiratory disease and mastitis^27,28^. However, IChek can be used measure multiple additional cytokines and other immune proteins including, TNF-α, another pro-inflammatory cytokine that has been shown to be critical in the immune response and pathology of bovine respiratory disease and bovine TB^29^. Anti-inflammatory cytokines will also be critical in future immune phenotyping work, as they are important in preventing exacerbated inflammatory responses.

An important outcome from the analysis conducted here is the identification of cattle with repeatable and divergent immune responses using IChek which could have important prognostic value for disease susceptibility. Multiple studies in cattle have associated cytokine responses to PAMPs with disease outcomes. For example, a study carried on bovine fibroblast stimulated with LPS, identified 4-low and 4-high IL-8 responders and selected them for *E. coli* intramammary infections^30^. It was found that not only was their IL-8 responses replicated in vivo, but that high responders exhibited higher tissue damage and had higher loss in milk yield. Another study by this group also found similar results with selecting 4-low and 4-high IL-8 responders after bovine fibroblast stimulations with Pam2CSK4 for *S. aureus* intramammary infections^31^. Although using a different cellular model, these studies show that capturing inter-individual variation in immune responses can identify immune phenotypes associated with disease susceptibility.

Cytokine responses between PAMPs were correlated and identified persistently low or high innate immune responders. Increased cell counts prior to stimulation were also found to correlate with cytokine responses, particularly monocytes and IL-6. Haematology is useful in diagnostic bovine medicine^20^, and our correlation analysis showed neutrophils and monocytes are positively associated and lymphocytes negatively associated with elevated inflammatory profiles. This was further illustrated by calculating correlations between cytokine responses and cell-to cell relative abundances, such as neutrophil:lymphocyte and neutrophil:monocyte ratios, which in human research have been linked with COVID-19 disease severity^32^, and monocyte:lymphocyte ratios, which have been previously linked to severity of tuberculosis infection^33^. High expression patterns of TLR receptors and subsequent increased responsiveness to TLR ligands in bovine granulocyte and monocyte cell populations are well characterized^34,35^, explaining why differences in these populations are linked with differential cytokine responses. However, this study shows that measuring cell abundances plays an important role when assessing variation in cytokine responses and identifies a possible mechanism of absolute cell counts prior to infections role in disease.

A major advantage of the IChek assay is its standardized approach to profiling immune responses between individuals and across time. Studies in humans have shown discordance in results between laboratories when using more labor-intensive cell culture stimulations compared to use of minimally manipulated whole blood, as such techniques run the risk of introducing artifacts due to manipulation and inter-operator variation^18,36,37^. Therefore, standardized technologies including IChek minimize technical variation and permit repeatable comparisons across cohorts, time points and between laboratories.

Previous work has also identified variation in adaptive immune responses^38^, and successfully associated low antibody-mediated immune responses with severe mastitis^39^. IChek has potential utility in tandem with such measures to comprehensively assess inter-individual variation in both innate and adaptive immune responses in cattle. IChek offers a low blood volume, low labor and low-cost standardized assay to profile the immune response, optimized in bovine whole blood for use with multiple disease relevant PAMPs. It is important to note that the flexible design could also be modified to use heat-killed bacteria or virus preparations for additional ex-vivo stimulations where relevant. Furthermore, this technology can be adapted for high-throughput immune phenotyping across cattle populations. The identification of prognostic or diagnostic immune response profiles would hold significant potential for reducing the burden of disease, our reliance on exogenous antibiotics and the potential transmission of zoonotic infections.

## Conclusion

There is urgent need for more detailed understanding of the immune response to infectious agents in livestock species in order to find alternatives to over reliance antibiotics and the emergence of antimicrobial resistance. Standardized immuno-assays including the IChek enable the comprehensive characterization of immune response phenotypes to disease-relevant antigens to ultimately identify immune signatures associated with disease resistance and susceptibility.

## Materials and methods

### Animals and sample collection

Holstein–Friesian calves were included in this study, ranging from ages 1 to 24 months for PAMP optimisation, and all ten animals were at approximately 24 months old for the longitudinal analysis. These animals were housed at the Teagasc Animal and Bioscience Research Centre, Trim, Co. Meath. Animals were kept in a standard agricultural environment with unrestricted access to food and water. They were TB negative prior to this study and were monitored closely for clinical signs of disease and required no medical interventions. The animals used in PAMP optimization had blood collected from them once. The ten animals used in the longitudinal analysis had blood collected from them at three timepoints over the span of 4 months, with 38 days between timepoint 1 and 2, and 42 days between timepoint 2 and 3.

### Blood collection and incubation using the ImmunoChek (IChek) assay

Whole blood was collected in Grenier Vacuette heparin and EDTA tubes (Cruinn Diagnostics Ltd) for the whole blood stimulation assay (WBSA) and for haematology analyses respectively. For whole blood stimulation (IChek), 1 mL of blood was diluted three-fold in a S-Monovette tubes (Sarstedt Ltd) pre-filled with 2 mL of RPMI media supplemented with 50 µg/mL streptomycin and 2.5 µg/mL amphotericin B (ThermoFisher Scientific). Pre-filled tubes contained either media only (null), or bacterial and viral PAMPs. Samples were subsequently placed in an incubator at 38.5 °C for 24 hr. Following incubation, supernatants were collected by centrifugation of tubes at 600*g* for 10 min, followed by aspiration of the cell culture supernatant.

### Haematology

Haematology profiles were established from blood collected in 6 mL EDTA coated vacutainers processed using the ADVIA 2120 haematology analyzer system to acquire total lymphocyte, monocyte, neutrophil, basophil and eosinophil cell numbers.

### PAMP concentrations

The selected PAMPs were chosen to represent major disease-relevant pathogens in cattle. Two serotypes of lipopolysaccharide (LPS) extracted from Gram-negative bacteria were used (*E. coli* O55:B5 and *E. coli* O11:B4, Sigma Aldrich), as potent activator of TLR4. LPS strains were tested at concentrations between 0.1 and 2 µg/mL, based on previous reports^35,40^. Pam3CSK4 (Pam3CysSerLys4) is a synthetic triacylated lipopeptide, chosen to mimic the response of lipopeptide from Gram-positive bacteria, and which is a potent activator of TLR1/2. Pam3CSK4 (InvivoGen) was tested at concentrations between 0.5 and 10 µg/mL, chosen based on previous reports^41,42^. Imidazoquinoline, also known as resiquimod (R848) (InvivoGen), was chosen to mimic the immune response to single stranded viral RNA (InvivoGen), and is a potent activator of TLR7/8. R848 was tested at concentrations between 0.1 and 2 µg/mL, chosen based on previous reports^43^.

### ELISA

Appropriate immune response readouts were chosen, for which reliable, validated assays were available commercially. The bovine IL-8 ELISA used to measure IL-8 levels in cell supernatants was carried out as previously reported^44^. The bovine IL-1β and IL-6 ELISAs (ThermoScientific) were performed as per manufacturer’s instructions. Protein concentrations expressed as the amount in 1 mL of blood and were calculated by multiplying by a dilution factor of 3. Technical replicate results were generated at the first time point and the means of these ELISA results were used to calculate the inter-individual variation.

### Statistical analysis

Data were analyzed using Prism software (v9.1) (www.graphpad.com) for one-way ANOVA with repeated measured and multiple comparisons used to test statistical significance for the PAMP concentration optimization. Normality of distribution was assessed prior to ANOVA analysis. Additionally, two-way ANOVA with repeated measures and multiple post-hoc comparisons was used to test statistical significance for PAMP responses across multiple timepoints. Coefficients of variation (CVs) were calculated for technical replicates to assess the technical variation of IChek, and between individuals at each timepoint to assess inter-individual variation, for each immune readout with the following formula: (SD/Mean)*100. Outlier analysis was completed for immune readouts and haematology data by calculating the upper and lower limits (upper limits: 3rd quartile (1.5 × IQR)) (lower limit: 1st quartile (1.5 × IQR)). Correlation analysis was completed using Pearson’s correlation.

### Ethical statement

All procedures described were conducted under ethical approval from the Teagasc Animal Ethics Commitee and experimental license from the Irish Health Products Regulatory Authority (licence numbers AE19132/P100 and AE19132/P099) in accordance with the Cruelty to Animals Act 1876 and the European Communities (Amendments of the Cruelty to Animals Act 1876) Regulations, 1994.

## Data Availability

The datasets analysed during the current study are available from the corresponding author on reasonable request.

## References

[1] Thacker TC, Palmer MV, Waters WR (2007). Associations between cytokine gene expression and pathology in *Mycobacterium bovis* infected cattle. Vet. Immunol. Immunopathol..

[2] Evans SE (2010). Stimulated innate resistance of lung epithelium protects mice broadly against bacteria and fungi. Am. J. Respir. Cell Mol. Biol..

[3] Gasteiger G (2017). Cellular innate immunity: An old game with new players. J. Innate Immun..

[4] Dinarello CA (2018). Overview of the IL-1 family in innate inflammation and acquired immunity. Immunol. Rev..

[5] Stojkovic B, Mullen MP, Donofrio G, McLoughlin RM, Meade KG (2017). Interleukin 8 haplotypes drive divergent responses in uterine endometrial cells and are associated with somatic cell score in Holstein–Friesian cattle. Vet. Immunol. Immunopathol..

[6] Chen L (2018). Inflammatory responses and inflammation-associated diseases in organs. Oncotarget.

[7] Banyer JL, Hamilton NH, Ramshaw IA, Ramsay AJ (2000). Cytokines in innate and adaptive immunity. Rev. Immunogenet..

[8] Duffy D (2014). Functional analysis via standardized whole-blood stimulation systems defines the boundaries of a healthy immune response to complex stimuli. Immunity.

[9] Duffy D (2018). Milieu intérieur: Defining the boundaries of a healthy immune response for improved vaccination strategies. Hum. Vaccines Immunother..

[10] Thomas S (2015). The Milieu Intérieur study—An integrative approach for study of human immunological variance. Clin. Immunol. Orlando Fla.

[11] Rodrigues KB (2020). Innate immune stimulation of whole blood reveals IFN-1 hyper-responsiveness in type 1 diabetes. Diabetologia.

[12] Duffy D (2020). Immune profiling enables stratification of patients with active TB disease or *M. tuberculosis* infection. Clin. Infect. Dis. Off. Publ. Infect. Dis. Soc. Am..

[13] Hadjadj J (2020). Impaired type I interferon activity and inflammatory responses in severe COVID-19 patients. Science.

[14] Jacobsen S, Toelboell T, Andersen PH (2005). Dose dependency and individual variability in selected clinical, haematological and blood biochemical responses after systemic lipopolysaccharide challenge in cattle. Vet. Res..

[15] Jacobsen S, Andersen PH, Toelboell T, Heegaard PMH (2004). Dose dependency and individual variability of the lipopolysaccharide-induced bovine acute phase protein response. J. Dairy Sci..

[16] Risalde MA (2011). Response of proinflammatory and anti-inflammatory cytokines in calves with subclinical bovine viral diarrhea challenged with bovine herpesvirus-1. Vet. Immunol. Immunopathol..

[17] Petry DB (2007). Differential immunity in pigs with high and low responses to porcine reproductive and respiratory syndrome virus infection. J. Anim. Sci..

[18] Duffy D (2017). Standardized whole blood stimulation improves immunomonitoring of induced immune responses in multi-center study. Clin. Immunol. Orlando Fla.

[19] O’Brien MB, McLoughlin RM, Meade KG (2020). Application of the TruCulture® whole blood stimulation system for immune response profiling in cattle. Vet. Immunol. Immunopathol..

[20] Roland L, Drillich M, Iwersen M (2014). Hematology as a diagnostic tool in bovine medicine. J. Vet. Diagn. Investig. Off. Publ. Am. Assoc. Vet. Lab. Diagn. Inc..

[21] Wood PR, Jones SL (2001). BOVIGAM: An in vitro cellular diagnostic test for bovine tuberculosis. Tuberc. Edinb. Scotl..

[22] Ackermann MR, Derscheid R, Roth JA (2010). Innate immunology of bovine respiratory disease. Vet. Clin. N. Am. Food Anim. Pract..

[23] Meager, A. & Wadhwa, M. An overview of cytokine regulation of inflammation and immunity. in *eLS* (American Cancer Society, 2013). 10.1002/9780470015902.a0024658.

[24] Foley C (2015). Integrated analysis of the local and systemic changes preceding the development of post-partum cytological endometritis. BMC Genom..

[25] Gabay C (2006). Interleukin-6 and chronic inflammation. Arthritis Res. Ther..

[26] Bochniarz M, Zdzisińska B, Wawron W, Szczubiał M, Dąbrowski R (2017). Milk and serum IL-4, IL-6, IL-10, and amyloid A concentrations in cows with subclinical mastitis caused by coagulase-negative staphylococci. J. Dairy Sci..

[27] Gao X (2019). Interleukin 8 and pentaxin (C-reactive protein) as potential new biomarkers of bovine tuberculosis. J. Clin. Microbiol..

[28] Hunt KM (2013). Mastitis is associated with increased free fatty acids, somatic cell count, and interleukin-8 concentrations in human milk. Breastfeed. Med. Off. J. Acad. Breastfeed. Med..

[29] Røntved CM (2000). Increased pulmonary secretion of tumor necrosis factor-alpha in calves experimentally infected with bovine respiratory syncytial virus. Vet. Immunol. Immunopathol..

[30] Kandasamy S, Green BB, Benjamin AL, Kerr DE (2011). Between-cow variation in dermal fibroblast response to lipopolysaccharide reflected in resolution of inflammation during *Escherichia coli* mastitis. J. Dairy Sci..

[31] Benjamin AL, Green BB, Hayden LR, Barlow JW, Kerr DE (2015). Cow-to-cow variation in fibroblast response to a toll-like receptor 2/6 agonist and its relation to mastitis caused by intramammary challenge with *Staphylococcus aureus*. J. Dairy Sci..

[32] Rizo-Téllez SA (2020). The neutrophil-to-monocyte ratio and lymphocyte-to-neutrophil ratio at admission predict in-hospital mortality in Mexican patients with severe SARS-CoV-2 infection (COVID-19). Microorganisms.

[33] Naranbhai V (2014). The association between the ratio of monocytes:lymphocytes at age 3 months and risk of tuberculosis (TB) in the first 2 years of life. BMC Med..

[34] Werling D, Hope JC, Howard CJ, Jungi TW (2004). Differential production of cytokines, reactive oxygen and nitrogen by bovine macrophages and dendritic cells stimulated with Toll-like receptor agonists. Immunology.

[35] Sohn EJ (2007). Bacterial lipopolysaccharide stimulates bovine neutrophil production of TNF-alpha, IL-1beta, IL-12 and IFN-gamma. Vet. Res..

[36] Mallone R (2011). Isolation and preservation of peripheral blood mononuclear cells for analysis of islet antigen-reactive T cell responses: position statement of the T-Cell Workshop Committee of the Immunology of Diabetes Society. Clin. Exp. Immunol..

[37] Moris P (2021). Whole blood can be used as an alternative to isolated peripheral blood mononuclear cells to measure in vitro specific T-cell responses in human samples. J. Immunol. Methods.

[38] Thompson-Crispi KA, Mallard BA (2012). Type 1 and type 2 immune response profiles of commercial dairy cows in 4 regions across Canada. Can. J. Vet. Res. Rev. Can. Rech. Veterinaire.

[39] Thompson-Crispi KA, Miglior F, Mallard BA (2013). Incidence rates of clinical mastitis among Canadian Holsteins classified as high, average, or low immune responders. Clin. Vaccine Immunol. CVI.

[40] Doherty R, O’Farrelly C, Meade KG (2013). Epigenetic regulation of the innate immune response to LPS in bovine peripheral blood mononuclear cells (PBMC). Vet. Immunol. Immunopathol..

[41] Kwok YH, Hutchinson MR, Gentgall MG, Rolan PE (2012). Increased responsiveness of peripheral blood mononuclear cells to in vitro TLR 2, 4 and 7 ligand stimulation in chronic pain patients. PLoS ONE.

[42] Hellman S, Hjertner B, Morein B, Fossum C (2018). The adjuvant G3 promotes a Th1 polarizing innate immune response in equine PBMC. Vet. Res..

[43] Jones GJ (2013). Immunisation with ID83 fusion protein induces antigen-specific cell mediated and humoral immune responses in cattle. Vaccine.

[44] Cronin JG, Hodges R, Pedersen S, Sheldon IM (2015). Enzyme linked immunosorbent assay for quantification of bovine interleukin-8 to study infection and immunity in the female genital tract. Am. J. Reprod. Immunol. N. Y. N 1989.

